# Convergence of patient- and physician-reported outcomes in the French National Registry of Facioscapulohumeral Dystrophy

**DOI:** 10.1186/s13023-021-01793-6

**Published:** 2022-03-02

**Authors:** Benoît Sanson, Caroline Stalens, Céline Guien, Luisa Villa, Catherine Eng, Sitraka Rabarimeriarijaona, Rafaëlle Bernard, Pascal Cintas, Guilhem Solé, Vincent Tiffreau, Andoni Echaniz-Laguna, Armelle Magot, Raul Juntas Morales, François Constant Boyer, Aleksandra Nadaj-Pakleza, Agnès Jacquin-Piques, Christophe Béroud, Sabrina Sacconi, The French FSHD registry collaboration group, Blandine Acket, Jean-Christophe Antoine, Shahram Attarian, Guillaume Bassez, Anne-Laure Bédat-Millet, Anthony Béhin, Rémi Bellance, Michela Bisciglia, Véronique Bombart, Rosalie Boitet, Pascale Bonnet, Françoise Bouhour, Célia Boutte, Brigitte Chabrol, Jean-Baptiste Chanson, Françoise Chapon, Ariane Choumert, Pauline Coignard, Jean-Yves Cornu, Benoît Daubail, Elisa De La Cruz, Léa Declerck, Capucine Delattre, Florence Demurger, Véronique Dulieu, Aurélie Duruflé, Fanny Duval, Florence Esselin, Teresinha Evangelista, Bruno Eymard, Anthony Faivre, Léonard Féasson, Xavier Ferrer, François Feuvrier, Olivier Flabeau, Mélanie Fradin, Alain Furby, Jérémy Garcia, Hélène Gervais-Bernard, Teresa Gidaro, Karima Ghorab, Marc Jeanpierre, Hubert Journel, Arnaud Lacour, Pascal Laforêt, Emmeline Lagrange, Valérie Layet, Gérard Leclaire, Jean-Luc Le Guiet, Gwenaël Le Guyader, François Leroy, France Leturcq, Nicolas Lévy, Sarah Léonard-Louis, Laurent Magy, Edoardo Malfatti, Marion Masingue, Gilles Mazaltarine, Dominique Ménard, Maud Michaud, Marie-Christine Minot-Myhié, Marie-Doriane Morard, Juliette Nectoux, Karine Nguyen, Julie Nicomette, Jean-Baptiste Noury, Sybille Pellieux, Laetitia Percebois-Macadré, Yann Péréon, Solange Perrin-Callot, Philippe Petiot, Sylviane Peudenier, Bénédicte Pontier, Florence Portet, Jean Pouget, Marguerite Preudhomme, Hélène Rauscent, Dimitri Renard, Audrey Riou, François Rivier, Emmanuelle Salort-Campana, Stéphane Schaeffer, Jean-Philippe Simon, Aurélie Siri, Marco Spinazzi, Tanya Stokovic, Juliette Svahn, François Tabaraud, Frédéric Taithe, Céline Tard, Christel Thauvin, Philippe Thoumie, Claire-Lise Tournier-Gervason, Christine Tranchant, Jon Andoni Urtizberea, Christophe Vial, Michel Vidaud, Fabien Zagnoli

**Affiliations:** 1 Université Côte d’Azur, Service Système Nerveux Périphérique & Muscle, Centre Hospitalier Universitaire de Nice, Nice, France; 2 Medical Affairs Department, AFM-Telethon, Evry, France; 3 Aix Marseille Univ, INSERM, MMG, Bioinformatics and Genetics, Marseille, France; 4 APHM, Hôpital Timone Enfants, Laboratoire de Génétique Moléculaire, Marseille, France; 5 Department of Neurology, Toulouse University Hospital, Toulouse, France; 6 Centre de Référence des Maladies Neuromusculaires AOC, Hôpital Pellegrin, CHU de Bordeaux, Bordeaux, France; 7 Centre de Référence des Maladies Neuromusculaires, Service de Médecine Physique et de Réadaptation, CHU de Lille, Lille, France; 8 Department of Neurology, APHP, Bicêtre University Hospital, Le Kremlin-Bicêtre, France; 9 French National Reference Center for Rare Neuropathies (NNERF), Le Kremlin-Bicêtre, France; 10 INSERM U1195 and Paris‐Saclay University, Le Kremlin-Bicêtre, France; 11 Referral Center for Neuromuscular Diseases Atlantique-Occitanie-Caraïbes, CHU Nantes, Nantes, France; 12 Département de Neurologie, CHU de Montpellier, Montpellier, France; 13 NMD Reference Center, Reims Champagne-Ardenne University Hospital, EA3797, Reims, France; 14 Centre de Référence des Maladies Neuromusculaires Atlantique-Occitanie-Caraïbes, FILNEMUS, Service de Neurologie, CHU d’Angers, Angers, France; 15 Centre de Référence des Maladies Neuromusculaires Nord/Est/Île-de-France, Service de Neurologie, Hôpitaux Universitaires de Strasbourg, Strasbourg, France; 16 Department of Clinical Neurophysiology, CHU Dijon Bourgogne, Dijon, France; 17 Institute for Research on Cancer and Aging of Nice (IRCAN), INSERM U1081, CNRS UMR 7284, Faculté de Médecine, Université Côte d’Azur (UCA), Nice, France

## Abstract

**Background:**

Facioscapulohumeral muscular dystrophy (FSHD) is among the most prevalent muscular dystrophies and currently has no treatment. Clinical and genetic heterogeneity are the main challenges to a full comprehension of the physiopathological mechanism. Improving our knowledge of FSHD is crucial to the development of future therapeutic trials and standards of care. National FSHD registries have been set up to this end. The French National Registry of FSHD combines a clinical evaluation form (CEF) and a self-report questionnaire (SRQ), filled out by a physician with expertise in neuromuscular dystrophies and by the patient, respectively. Aside from favoring recruitment, our strategy was devised to improve data quality. Indeed, the pairwise comparison of data from 281 patients for 39 items allowed for evaluating data accuracy. Kappa or intra-class coefficient (ICC) values were calculated to determine the correlation between answers provided in both the CEF and SRQ.

**Results:**

Patients and physicians agreed on a majority of questions common to the SRQ and CEF (24 out of 39). Demographic, diagnosis- and care-related questions were generally answered consistently by the patient and the medical practitioner (kappa or ICC values of most items in these groups were greater than 0.8). Muscle function-related items, i.e. FSHD-specific signs, showed an overall medium to poor correlation between data provided in the two forms; the distribution of agreements in this section was markedly spread out and ranged from poor to good. In particular, there was very little agreement regarding the assessment of facial motricity and the presence of a winged scapula. However, patients and physicians agreed very well on the Vignos and Brooke scores. The report of symptoms not specific to FSHD showed general poor consistency.

**Conclusions:**

Patient and physician answers are largely concordant when addressing quantitative and objective items. Consequently, we updated collection forms by relying more on patient-reported data where appropriate. We hope the revised forms will reduce data collection time while ensuring the same quality standard. With the advent of artificial intelligence and automated decision-making, high-quality and reliable data are critical to develop top-performing algorithms to improve diagnosis, care, and evaluate the efficiency of upcoming treatments.

**Supplementary Information:**

The online version contains supplementary material available at 10.1186/s13023-021-01793-6.

## Background

Facioscapulohumeral Dystrophy (FSHD) is one of the most common dystrophies in adults. The prevalence of the disease has been reported ranging from ~ 1:8000 to ~ 1:15,000 in the US [1, 2], ~ 1:8000 in the Netherlands [3], and ~ 1:20,000 in Italy [4] and the UK [5, 6]. No epidemiological study has been performed in France yet, but by conservative standards, it can be estimated that at least 3500 people carry the disease.

FSHD is characterized by progressive asymmetric muscle weakness, with early involvement of facial muscles, progressive weakness and atrophy of scapular and humeral muscles, and later involvement of the trunk and lower extremities. The disease shows significant inter- and intra-familial clinical variability in terms of progression and severity. Disease onset is usually before the second decade; early onset is associated with faster progression and higher severity as most wheelchair-bound FSHD patients have had childhood onset of the disease [7, 8]. These severe FSHD patients are more prone to develop an extra muscular complication of the disease, such as central nervous system involvement [9, 10], retinal telangiectasia [11], and hearing impairment [12].

FSHD is associated with epigenetic derepression of D4Z4 repeats on chromosome 4q. The common form FSHD type 1 (FSHD1; ~ 95% of patients) is associated with a pathogenic contraction of D4Z4 repeat units (RUs; 1–10). Patients with the rare form FSHD type 2 (FSHD2) have more than 10 D4Z4 RUs combined with defects in D4Z4 chromatin repressors, mostly *SMCHD1* gene mutations [13]. It has recently been suggested that the two types of the disease correspond to a genetic and epigenetic continuum [14]. In both FSHD types, the aberrant expression of the D4Z4-encoded *DUX4* gene has been proposed to cause the disease through a toxic gain-of-function mechanism [15].

Genetic and clinical heterogeneity [16] in FSHD may complicate diagnosis and proper genetic counseling, and prevent the development of clinical, biological and patient-reported outcome measures (PROMs) to evaluate disease severity and progression, and the efficacy of therapeutic strategies.

Until recently, data on FSHD were scarce. To ease data collection on FSHD, national registries have been, or are being, set up in Europe, Northern America, Egypt, Australia, and New Zealand [6, 8, 17–19]. While efforts have been made to ensure a common data set [20], the objectives of such databases are multiple. For instance, the Italian registry [18] predominantly aims at gaining novel insights into the natural history of the disease. In contrast, the US [8], UK [6], and New Zealand [17] registries have been designed as tools to ease the enrollment of patients in clinical trials and studies. The French FSHD registry has been collecting data since 2013, and purposed both with a better understanding of the natural course of FSHD and the facilitation of clinical trials, e.g. by identifying PROMs or tailoring eligibility criteria.

Data are often gathered through clinical evaluation, which relies on the active participation of medical practitioners. Alternatively, some databases aggregate patient-reported data. However, the ability of FSHD patients in collecting data on their disease has never been investigated. Most registries use a single questionnaire, filled out either by the physician or by the patient. Except for the US registries, where a minority of questions are addressed to the clinician, all registries collect data from a single source. As it is unlikely that a single rater best assesses all relevant registry items, the French FSHD registry was designed to gather information from both the patient with a self-report questionnaire (SRQ) and the neurologist with a clinical evaluation form (CEF) [19]. The objective was to not only reach a greater population, but also to assess and improve data accuracy and quality, which is critical in order to harmonize and share data at the international level, thus enabling machine-learning (ML) and AI approaches.

This study evaluates the concordance between SRQs and CEFs used in the French FSHD registry in a cohort of 281 patients to optimize these forms for high-quality data collection.

## Patients and methods

### Patient cohort

**Fig. 1:**
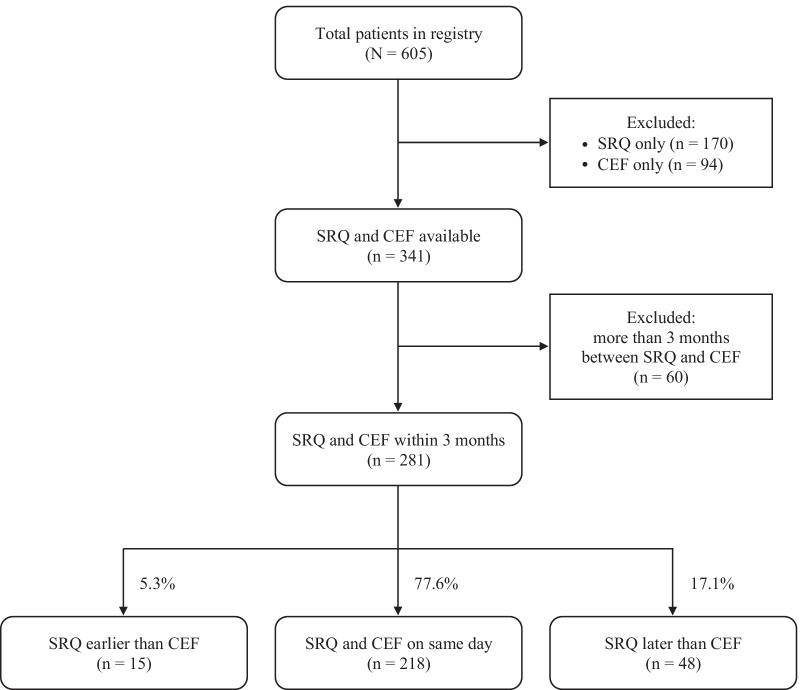
Patient selection process in the analysis. The distribution of selected patients, i.e., having both a self-report questionnaire (SRQ) and a clinical evaluation form (CEF) filled within 3 months at the time of analysis, is shown according to the order of completion of forms

Data were collected using predesigned forms: an SRQ and a CEF (available on the registry website, www.fshd.fr) [19] completed by the patient and the neurologist, respectively. FSHD1 patients of the French National Registry of FSHD were included in the analysis, provided both forms were available. Additionally, the two forms were to be completed within a period of 3 months, which was deemed short enough to prevent a significant evolution of clinical signs and symptoms between the two assessments. Among the 605 patients included in the registry at the time of the analysis (September 2017), 281 patients were selected. Fig 1 shows the flowchart of the selection process. Signed informed consent was obtained from each patient before any data collection. The relevant national ethics committees approved the registry, namely the French data protection authority (CNIL; Authorization Number 912291) and the French advisory committee on data processing in health research (CCTIRS; Favorable Opinion Number 12.004bis).

### Data collected

**Fig. 2:**
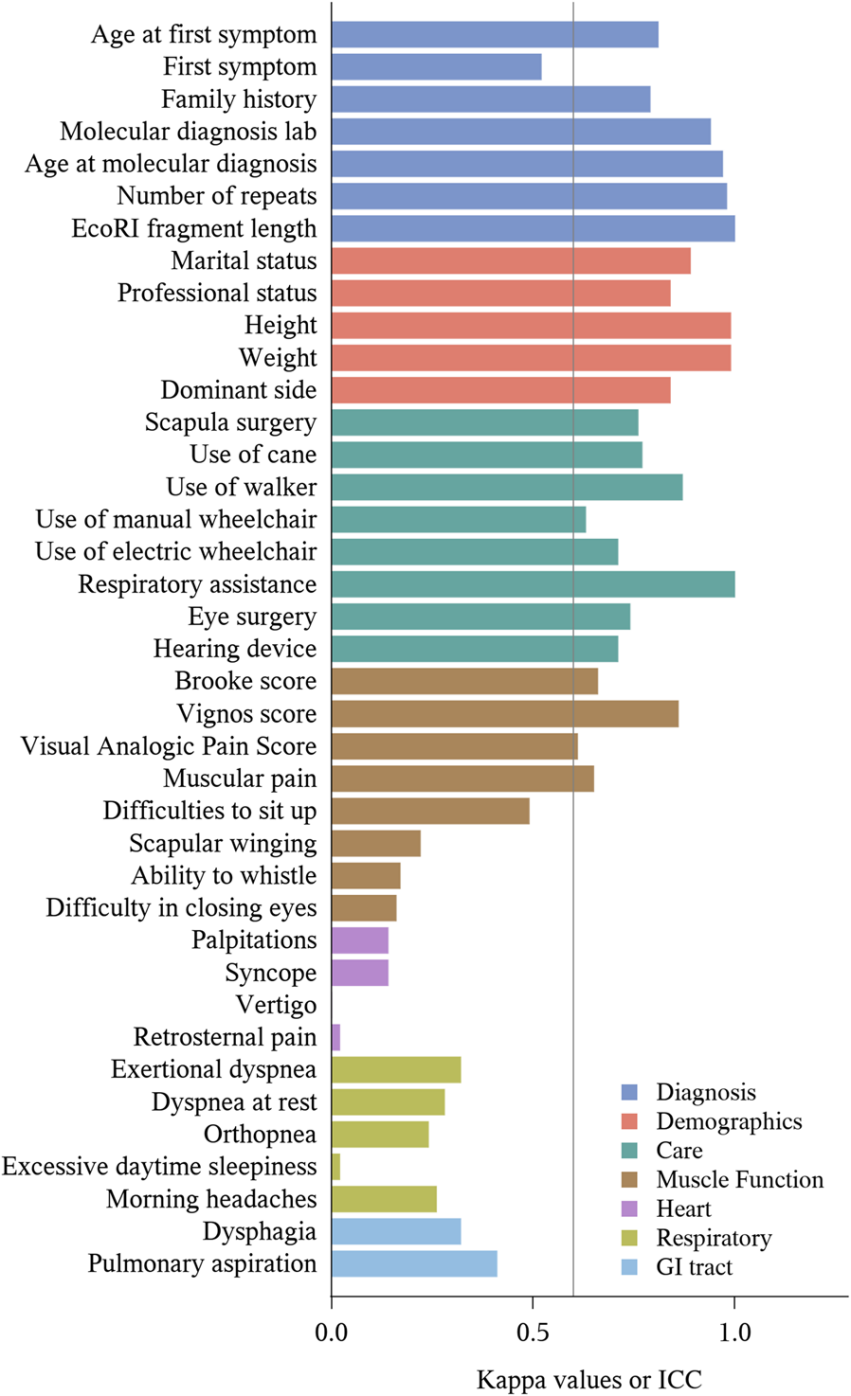
Agreement (in Kappa value or ICC) between SRQ and CEF answers to items used in the statistical comparison. The line at Kappa/ICC = 0.6 represents the cut-off value beyond which agreement is deemed good

Items collected in both the SRQ and the CEF were analyzed to evaluate internal consistency between the two raters (patient and clinician). The forms enclose 42 mutual items, out of which 39 could be statistically assessed in a relevant manner (Fig. 2 and Table 1). The 39 compared items are divided into seven sections: diagnosis, demographics, muscle function, care; as well as heart, respiratory and GI symptoms. It should be noted that the SRQ asked for a self-evaluation of muscle function. Indeed, patients were proposed to self-grade their arm and leg function using reformulated Brooke [21] and Vignos [22] scales; physicians usually perform such scoring. Patients were also asked to self-evaluate facial involvement through yes/no questions such as "Are you able to whistle?" or "Do you have difficulty closing your eyes?".

**Table 1:** Inter-rater agreement, evaluated as a percentage agreement, and as a kappa value or ICC, for the 39 items common to the SRQ and CEF used in the statistical comparison; class sizes are provided and items are grouped by sections

Section	Item	n	PA (%)	Kappa [95% CI]	ICC [95 % CI]
Diagnosis	Age at first symptom	236	–	–	0.81 [0.76–0.85]
	First symptom	255	0.68	0.52 [0.44–0.60]	–
	Family history	266	0.90	0.79 [0.72–0.87]	–
	Molecular diagnosis lab	253	0.97	0.94 [0.90–0.98]	–
	Age at molecular diagnosis	236	–	–	0.97 [0.96–0.98]
	Number of repeats	219	–	–	0.98 [0.97–0.98]
	EcoRI fragment length	8	–	–	1.00 [ – ]*
Demographics	Marital status	270	0.93	0.89 [0.84–0.93]	–
	Professional status	272	0.89	0.84 [0.79–0.89]	–
	Height	271	–	–	0.99 [0.99–0.99]
	Weight	266	–	–	0.99 [0.99–0.99]
	Dominant side	268	0.96	0.84 [0.75–0.93]	–
Care	Scapula surgery	276	0.97	0.76 [0.61–0.92]	–
	Use of cane	281	0.93	0.77 [0.67–0.86]	–
	Use of walker	281	0.98	0.87 [0.77–0.96]	–
	Use of manual wheelchair	281	0.92	0.63 [0.49–0.77]	–
	Use of electric wheelchair	281	0.93	0.71 [0.58–0.83]	–
	Respiratory assistance	19	1.00	1.00 [1.00–1.00]	–
	Eye surgery	40	0.88	0.74 [0.54–0.95]	–
	Hearing device	30	0.87	0.71 [0.46–0.97]	–
Muscle function	Brooke scale	254	–	–	0.66 [0.58–0.72]
	Vignos scale	250	–	–	0.86 [0.82–0.89]
	Visual Analog Pain Score (VAPS)	191	–	–	0.61 [0.51–0.69]
	Muscular pain	270	0.89	0.65 [0.53–0.76]	–
	Difficulties to sit up	263	0.80	0.49 [0.37–0.61]	–
	Scapular winging	241	0.86	0.22 [0.06–0.38]	–
	Ability to whistle	271	0.55	0.17 [0.10–0.25]	–
	Difficulties in closing eyes	272	0.46	0.16 [0.10–0.21]	–
Heart	Palpitations	281	0.74	0.14 [0.04–0.23]	–
	Syncope	281	0.92	0.14 [-0.04–0.32]	–
	Vertigo	281	0.77	0.00 [0.00–0.00]	–
	Retrosternal pain	281	0.81	0.02 [-0.05–0.08]	–
Respiratory	Exertional dyspnea	281	0.71	0.32 [0.21–0.43]	–
	Dyspnea at rest	281	0.92	0.28 [0.06–0.49]	–
	Orthopnea	281	0.94	0.24 [0.01–0.47]	–
	Excessive daytime sleepiness	281	0.79	0.02 [-0.07–0.12]	–
	Morning headaches	281	0.88	0.26 [0.10–0.42]	–
GI tract	Dysphagia	281	0.85	0.32 [0.16–0.47]	–
	Pulmonary aspiration	281	0.85	0.41 [0.27–0.55]	–

*n* number of observations, *PA* percentage of agreement, *ICC* intraclass correlation coefficient, CI confidence interval, *VAPS* visual analog pain scale

* *CI* not computed because the number of observations was too small

### Statistical analysis

For qualitative items, an inter-rater reliability analysis using Cohen's kappa statistic was performed. The definition is $K=\frac{{p}_{o}-{p}_{e}}{1-{p}_{e}},$ where p_o_ is the relative observed agreement among raters and p_e_ is the relative agreement among raters expected by chance. The interpretation was performed using the Landis and Koch guidelines [23], while considering the known limitations of the kappa statistic, such as the prevalence of observations and heterogeneous margins [24, 25].

For quantitative items, intraclass correlation coefficients (ICC) were used. We considered a "two-way random-effects" model as we planned to generalize our reliability results to any rater possessing the same characteristics as those selected (patient or clinician) and a "single rater/measurement" type as we planned to use the measurement from a single rater as the basis of the actual measurement [26]. The degree of consistency is $\hat{\rho }=\frac{MS_{R}-MS_{E}}{MS_{R}+MS_{E}},$ where MS_R_ and MS_E_ are the mean squares for rows and error, respectively. The ICC interpretation was performed according to the criteria used for that of the kappa coefficient.

The statistical analysis was performed using the SAS software, version 9.4 (SAS Institute Inc., Cary, NC, USA).

### Subgroup analyses

The same analysis was performed separately on data split into two groups according to a series of parameters: gender, age (< 65 years vs. ≥ 65 years), education level (pre- vs. post- French secondary education degree), and disease severity (Clinical Severity Score [27] < 6 vs. ≥ 6).

## Results

### Description of the cohort

The cohort consisted of 131 women and 150 men with a mean age of 54.8 ± 16.0 years (Table 2). With a number of RUs ranging from 2 to 10 and 11% of non-ambulatory patients, the cohort can be deemed representative of the general FSHD population.

**Table 2:** Statistics description of the cohort

Characteristic	n = 281
Gender	
Male	150 (53.4%)
Female	131 (46.6%)
Age (years)	
Mean ± SD	54.8 ± 16.0
Range	21–96
D4Z4 repeat array size (units)	2–10
Ability to walk	
Yes	250 (89.0%)
No	31 (11.0%)

*SD* standard deviation

As shown in Fig. 1, the SRQ and CEF were filled out on the same day in most cases (77.6%). SRQs were filled out later than CEFs more often than earlier (17.1% vs. 5.3%, respectively). The mean time between the filling out of two forms was 0.62 days ± 11.82 days (data not shown).

From this point on, all the following results pertain to items with an answer in both the SRQ and the CEF. Among the response pairs, the age at onset, completed in both forms for 84% of patients (236 out of 281), varied from 0 to 75 years in the SRQ, and from 1 to 73 years in the CEF (Table 3). The  first symptoms appeared on average in the late twenties, with a mean age of 28.6 ± 17.4 years in the SRQ and 26.9 ± 16.6 years in the CEF. They were primarily localized in the proximal end of the superior limb: 51% and 55%, according to patients and neurologists, respectively, out of 255 answer pairs (Table 3).

**Table 3:** Clinical diagnosis data recorded by form type

Characteristic	Self-report questionnaire	Clinical evaluation form
Age at onset (n = 236)		
Mean ± SD Range (years)	28.6 ± 17.4 0–75	26.9 ± 16.6 1–73
First symptom (n = 255)		
Asymptomatic patient	2 (0.8%)	2 (0.8%)
Facial involvement	41 (16%)	38 (15%)
Proximal upper limb involvement	130 (51%)	139 (55%)
Distal upper limb involvement	2 (0.8%)	7 (2.7%)
Proximal lower limb involvement	41 (16%)	27 (11%)
Distal lower limb involvement	10 (3.9%)	25 (9.8%)
Other symptom	28 (11%)	17 (6.7%)

### Comparative analysis of patient and physician assessments

For each of the 39 analyzable items, the consistency between the responses in the two types of forms was evaluated (Table 1 and Fig. 2). Specific results per item category are given below.

### Diagnosis

Most items related to diagnosis, i.e. related to first symptoms or genetics, showed excellent consistency between patient and physician reporting (kappa value higher than 0.8). Interestingly, the age at first symptoms was highly consistent (ICC equal to 0.81). Most genetic-related items, namely the laboratory where the analysis was performed, the age at the time of molecular diagnosis, the number of RUs, and the EcoRI fragment length, were nearly identical in form pairs (kappa value or ICC of 0.94, 0.97, 0.98 and 1, respectively). Moreover, the consistency of the item "family history" was also relatively high (kappa of 0.79). However, the description of the first symptom is an outlier in this section as it was associated with a medium consistency (kappa of 0.52). The prevalence of all items in this section was between 219 and 266, except for the EcoRI fragment length, which was completed in only eight form pairs. It is also worth noting that the number of RUs was answered significantly more often by the physician (255; data not shown) than the patient (219).

### Demographics

As expected, all items in the demographic section showed a good agreement between the patient and the physician (kappa or ICC greater than or equal to 0.84; Table 1). Height and weight, in particular, had a very high agreement (ICC of 0.99). Though slightly lower, dominant side, and marital and employment statuses also showed excellent consistency (kappa values of 0.84, 0.89, and 0.84, respectively). With a prevalence greater than or equal to 266 (out of a cohort of 281), demographic items were reported in nearly all form pairs.

### Medical care

The data indicated a good to excellent agreement for all items related to surgical procedures and the use of a medical device. Indeed, the items related to scapula and eye surgery, and the use of a hearing aid and most walking devices listed in the forms (cane, manual and electric wheelchairs), showed a good agreement (kappa values between 0.63 and 0.77). Furthermore, the items "use of a walker" and "respiratory assistance" showed excellent agreement (kappa values of 0.87 and 1, respectively). The prevalence of all items in this section was very high, except for "respiratory assistance", "hearing aid" and "eye surgery" (19, 30, and 40, respectively). The only caveat is that a prevalence of 281 was systematically observed for the 15 items related to multiple-choice questions where "no" was not a proposed choice. Indeed, owing to the data structure, the absence of an answer was then indistinguishable from "none" or "no".

### Muscle function

The prevalence of all items in this section was greater than or equal to 241, except for that related to the visual analog pain score (VAPS) with 191 (see Table 1). The distribution of agreements was very diffuse. On the one hand, the agreement between patients and physicians regarding body motricity was good to very good, except for the item "difficulties to sit up", which showed a medium agreement. Indeed, the item related to the Vignos score, which evaluates the lower extremity function on a scale from 1 to 10, 1 being the least severe involvement, showed a very high agreement (ICC of 0.86). Additionally, the Brooke score, which is the upper extremity counterpart of the Vignos score (on a scale from 1 to 6), as well as the items "muscular pain" and "Visual Analog Pain Score (VAPS)", yielded good agreements (kappa value or ICC between 0.61 and 0.66). On the other hand, patients and physicians mostly disagreed when assessing facial motricity ("difficulty in closing eyes" and "ability to whistle") and "scapular winging" (kappa values between 0.16 and 0.22).

Interestingly, the item "difficulty in closing eyes" was predominantly answered negatively by patients (76% of SRQs; Table 4) but positively by physicians (72% of CEFs). It is striking that most of the related discordance (50% of answer pairs) was associated with patients answering "no" in the SRQ but having a counterpart "yes" in the matching CEF. The opposite situation ("yes" in the SRQ and "no" in the CEF") was observed in only 1.1% of cases. The item "ability to whistle" showed a similar discrepancy, although in reverse (a majority of "yes" in the SRQ matched by a "no" in the CEF) and with more balanced associated SRQ answers. The item "scapular winging" exhibited a similar trend but to a more limited extent. Although the agreement was low, the percentage of agreement was still relatively high (86%; Table 1).

**Table 4:** Breakdown of response pairs, in the CEF and SRQ, to four discordant items

Item	SRQ answer	CEF answer			
Difficulty in closing eyes		Uncertain	No	Yes	Total
	Uncertain	0 (0.0%)	0 (0.0%)	1 (0.4%)	1 (0.4%)
	No	8 (2.9%)	65 (24%)	135 (50%)	208 (76%)
	Yes	0 (0.0%)	3 (1.1%)	60 (22%)	63 (23%)
	Total	8 (2.9%)	68 (25%)	196 (72%)	272 (100%)
Ability to whistle		Uncertain	No	Yes	Total
	Uncertain	0 (0.0%)	2 (0.7%)	0 (0.0%)	2 (0.7%)
	No	1 (0.4%)	114 (42%)	7 (2.6%)	122 (45%)
	Yes	2 (0.7%)	109 (40%)	36 (13%)	147 (54%)
	Total	3 (1.1%)	225 (83%)	43 (16%)	271 (100%)
Winged scapula			No	Yes	Total
	No		6 (2.5%)	30 (12%)	36 (15%)
	Yes		3 (1.2%)	202 (84%)	205 (85%)
	Total		9 (3.7%)	232 (96%)	241 (100%)
Vertigo			No	Yes	Total
	No		216 (77%)	0 (0.0%)	216 (77%)
	Yes		65 (23%)	0 (0.0%)	65 (23%)
	Total		281 (100%)	0 (0.0%)	281 (100%)

*SRQ* self-report questionnaire, *CEF* clinical evaluation form

One of the aforementioned items is formulated as an ability while the two others as a difficulty. The disagreements observed in the facial motricity-related and "scapular winging" items were thus similar: in most cases, when answers did not match, the observed disagreement was one-way. More specifically, the absence of a report of FSHD-specific symptoms in the CEF was rarely matched with a report in the SRQ. On the contrary, the report of such symptoms in the CEF was comparably associated with either a report or an absence thereof in the SRQ.

### Signs and symptoms not specific to FSHD

The agreement of items related to signs and symptoms not specific to FSHD, included in the analysis, ranged from very poor to medium. A poor to very poor agreement (kappa value between 0 and 0.32) was observed for all items related to heart and respiratory signs and symptoms. GI tract-related items "dysphagia" and "pulmonary aspiration" showed a low to medium agreement (kappa value of 0.32 and 0.41 respectively). In contrast, the prevalence was very high and equal to 281 for all items, with the caveat mentioned above.

The item "vertigo and dizziness" showed a peculiar phenomenon, with patients much more prone to report symptoms than physicians. Strikingly, no positive answer was recorded in the CEFs, although 23% of SRQs recorded "yes" (Table 4). A similar observation was made with "retrosternal pain", where the CEFs of nearly all patients (99%) reported a "no" but 18% positive answers were found in the SRQs (data not shown). Hence, positive responses in the CEF were scarcely matched with a negative answer in the SRQ, i.e. the mismatch happened one way, not the other, suggesting that specific symptoms were not necessarily wrongly identified but may have been overlooked by physicians or overestimated by patients. The two items mentioned above displayed the lowest agreement in the analysis (kappa values of 0 and 0.02% for vertigo and retrosternal pain, respectively).

Overall, symptoms reported in the SRQ were not systematically reported by the neurologist. The symptoms most frequently unparalleled by the physician in the CEF were vertigo, retrosternal pain, and daytime sleepiness. The unparalleled reporting of symptoms between form types was further analyzed by center, yielding no significant difference (data not shown).

### Summary

Items corresponded to either quantitative or qualitative variables. All quantitative variables yielded high agreement (ICC greater than 0.6). Qualitative variables did not display a specific trend in agreement (kappa values scattering the whole range from 0 to 1).

### Effect of age, sex, education level, and disease severity on rates of agreement

The cohort was split into two groups according to a predefined cut-off in each category to assess the effects of age, sex, education level, and disease severity. The statistical comparison performed on these groups did not significantly differ relative to the general cohort (Additional file 1: Figure S1, Additional file 2: Figure S2, Additional file 3: Figure S3, Additional file 4: Figure S4, Additional file 5: Figure S5, Additional file 6: Figure S6, Additional file 7: Figure S7, Additional file 8: Figure S8).

## Discussion

### Cohort representativeness

In this study, we compared data from SRQs and CEFs. Data were analyzed only if both forms were available and had been filled out within a 3-month period, to mitigate variations due to disease evolution. Overall, data from the whole registry and our selected cohort of 281 patients were consistent with what was observed in epidemiological studies [1–6]. Indeed, 95.01% of patients in the French registry had FSHD1 at the time of analysis, perfectly reflecting the finding that type 1 represents at least 95% of cases [28]. Besides, in the selected cohort, the first symptoms appeared on average in the late twenties, as previously described [29]. The consistency of the cohort with preexisting epidemiological data can be further established. For instance, the initial symptom most often reported in the French registry, and the cohort, was an involvement of the proximal upper limb muscles, as described in several epidemiological studies [6]. Another evidence can be found in the broad distribution of ages (21–99), and nearly complete FSHD1-compatible range of RUs (2–10) observed in the study cohort.

### Concordance of patient- and physician-reported data

The comparison of the answers provided by patients and physicians on 39 items allowed assessing the inter-rater reliability of the data reported. To our knowledge, it is the first time that the agreement of patient- and physician-reported data has been evaluated in a neuromuscular disease registry. The present study was made possible by the dual data collection strategy set forth in the French FSHD registry.

Our results showed that patients and physicians agreed in most cases. Indeed, a majority of items analyzed (24 out of 39) showed good to excellent agreement. Most questions found in the demographic, diagnosis, muscle function, and care sections were answered identically by the physician and the patient. It follows that the corresponding PROMs are as trustworthy as the assessments made by the physician, and the related questions thus need not necessarily be asked to the latter to collect a full dataset on a patient at a given time. As the medical consultation time is limited, it is highly desirable to optimize the CEF by either shortening it as much as possible or replacing superfluous items with assessments requiring medical expertise. The paramount importance of patients in contributing to the registry through self-reporting data is herein evidenced.

### Patients and physicians disagree on symptoms

Patients and physicians gave discordant answers regarding most signs and symptoms. The structure of the discordance suggests that patients tend to ignore or minimize the impairments the FSHD-specific symptoms are related to, which is in accord with our experience and has been described in patients with oculopharyngeal muscular dystrophy [30]. However, symptoms related to comorbidities showed a different behavior. In contrast to FSHD-specific signs, patients tended to report symptoms not specific to FSHD more readily than physicians. Some symptoms may require a trained specialist to be recognized but are more likely to be overlooked by said specialist when filling out a time-consuming comprehensive form. Therefore, no single data source should be privileged in this case. Even though the physician answers may be more accurate, the patient information is more complete. Comorbidity-related symptoms should thus be collected in both forms.

### Limitations and biases

Although concordant when answered in both forms, several items, such as genetic information, were moderately to scarcely reported by patients, likely owing to their inherent technicality. Such items are therefore best left to the physician. It is worth noting that the number of RUs and fragment length are two sides of the same coin; either information was sufficient, thereby deterring physicians from providing redundant information. The fragment length may thus be removed from future forms.

The order in which SRQs and CEFs were completed likely influenced the answers provided in either form. The influence of each rater on the other thus cannot be ruled out. Furthermore, potential help from the staff in completing either form probably drove up concordance. However, although representing a statistical bias in the present study, such an effect actually underscores the effectiveness of using both patient- and physician-reported forms by helping collect complete and reliable datasets.

Data reported in the CEFs were carefully monitored, except when related to symptoms. Moreover, physicians are trained to assess outcome measures. In this light, we could reasonably, albeit roughly, assume the reliability of objective physician-reported data. In the following, for practical reasons, we thus equated inter-rater concordance to patient reliability regarding relevant items.

### Optimization of data collection forms

The concordance of responses is highly dependent on the nature of the question and the collection modality. Our analysis showed that data can reliably be collected directly by the patient, provided it is straightforward, objective, or quantitative. In particular, the surprisingly good agreements of the Vignos and Brooke scales showed that patients could accurately answer detailed, technical questions. In contrast to the description of symptoms, the lower and upper limb functional assessment scales are defined in simple words. The general higher consistency of quantitative variables, therefore, drives us to employ quantifiable items whenever possible. The formulation of questions is also essential. Notably, medical jargon should be avoided in SRQs.

The availability of physicians is a limiting factor when collecting data for a registry. This is also true, to a lesser extent, for patients who are generally asked to fill many questionnaires when visiting their FSHD doctor. The registry questionnaires must therefore be as short as possible. However, as the natural history and causes of FSHD are still largely being explored, relevant questions are many. To minimize the burden for both raters, we propose not to ask clinical or technical questions to patients unless it has an objective or quantifiable aspect, such as the Vignos and Brooke scales.

Conversely, all questions that do not specifically require medical training can be left to the patient. Nevertheless, a number of such questions should be asked in both forms as an internal control to further assess the reliability of answers provided and pursue the optimization of the forms. Furthermore, it can help collect data that physicians will not provide for lack of time, e.g. data related to non-FSHD symptoms, even though the reliability of which cannot be assumed (with the limitations mentioned above) as positively as that of objective data. Besides, both forms are not available for all patients. Objective or quantifiable items may thus be retained optionally in the CEF (items related to signs and symptoms were already optional in the SRQ [19]).

Considering the reliability of most patient-reported data and the associated limitations, and given that no effect of gender, age, education level, or disease severity was observed, the registry forms have been modified to increase concordance and efficiency. In particular, the formulation of questions has been optimized; patient feedback helped in this task. Since we established that the registry can rely more on patient-reported data, the CEF contents were revised to bring the focus on discordant items.

### Patient-related outcome measures are key to further research

The present study validates and reinforces the French registry philosophy: recording the patient and physician complementary visions is invaluable to warrant the data quality expected to lead relevant statistical analysis, in particular based on ML techniques. The natural course of FSHD is highly variable and predicting disease outcomes is not yet achievable. The so-called ReSolve clinical study (NCT03458832) has been set up as a way to identify novel PROMs and expand knowledge on the natural history of FSHD [31]. Registries are instrumental in collecting PROMs [6, 32, 33] as they are an increasingly important key feature of clinical trials [34]. The combination of patient- and physician-reported data in the French FSHD registry will be a significant asset in gathering the data necessary to define the objectives and outcome measures, and fine-tune the eligibility criteria, of future clinical trials.

### Building predictive models

Alternatively, and complementarily, applying ML on the registry data could help better characterize the stages of disease progression and make individualized predictions. Furthermore, it is believed that AI will play a fundamental role in finding treatments for rare diseases [35]. ML has recently started to be implemented in the diagnostic process of some neuromuscular diseases, by improving the analysis of electromyograms [36] or MRI patterns [37]. It is also used in the context of autoimmune diseases, including neuromuscular disorders such as myasthenia gravis, to help predict the disease outcomes [38]. By pooling national datasets, thereby vastly increasing the data available, the global FSHD project [39] would greatly potentiate the efficiency of AI analyses. The hindrance towards establishing a predictive FSHD model may be more of an administrative and regulatory nature than of a scientific one.

## Conclusions

This study showed that patient-reported data are as reliable as physician-made assessments on condition that they are objective or quantifiable, which includes, surprisingly, the Brooke and Vignos scores. This finding helped optimize the forms used in the French FSHD registry, which will be a key resource for designing future therapeutic trials and improving standards of care, in part through the development of PROMs. As the outcome of telemedicine consultations in France, and other parts of the world, in the wake of the Covid-19 pandemic showed, the identification of reliable PROMs is at the heart of the future of medical practice. Achieving high-quality FSHD data is thus all the more important. However, extracting relevant new information, through e.g. ML techniques, may require pooling out resources from several registries. In this view, performing the same comparative analysis on data from patient- and clinician-based registries would be an interesting follow-up study and could benefit the harmonization registries require to bring out relevant and usable PROMs.

## Supplementary Information

**Additional file 1. Figure S1**: Agreement (in Kappa or ICC values) between item answers in the SRQ and the CEF in the younger subgroup of the cohort (< 65 years; N = 209).

**Additional file 2. Figure S2**: Agreement (in Kappa or ICC values) between item answers in the SRQ and the CEF in the older subgroup of the cohort (≥ 65 years, N = 209).

**Additional file 3. Figure S3**: Agreement (in Kappa or ICC values) between item answers in the SRQ and the CEF in the subgroup associated with less severe forms of the disease (CSS < 6, N = 72).

**Additional file 4. Figure S4**: Agreement (in Kappa or ICC values) between item answers in the SRQ and the CEF in the subgroup associated with more severe forms of the disease (CSS ≥ 6; N = 165).

**Additional file 5. Figure S5**: Agreement (in Kappa or ICC values) between item answers in the SRQ and the CEF in the subgroup associated with men.

**Additional file 6. Figure S6**: Agreement (in Kappa or ICC values) between item answers in the SRQ and the CEF in the subgroup associated with women.

**Additional file 7. Figure S7**: Agreement (in Kappa or ICC values) between item answers in the SRQ and the CEF in the subgroup associated with pre- French secondary education degree.

**Additional file 8. Figure S8**: Agreement (in Kappa or ICC values) between item answers in the SRQ and the CEF in the subgroup associated with post- French secondary education degree.

## Data Availability

Data are accessible on the registry website, http://www.fshd.fr, through log-in credentials for protecting personal privacy and proprietary information. However, the aggregated data supporting the findings of this study can be obtained upon reasonable request to the French FSHD registry steering committee.

## Abbreviations

AFM-Téléthon: French Muscular Dystrophy Association
AI: Artificial intelligence
CCTIRS: French Advisory Committee on data processing in health research
CEF: Clinical evaluation form
CNIL: French Data Protection Authority
FSHD: Facioscapulohumeral muscular dystrophy
FSHD1: FSHD type 1
FSHD2: FSHD type 2
ICC: Intra-class coefficient
ML: Machine learning
PROM: Patient-reported outcome measure
*SMCHD1*: Structural maintenance of chromosomes flexible hinge domain containing 1
SRQ: Self-report questionnaire
VAPS: Visual Analog Pain Scale
