# Supplementary figures and images for: Convergence of patient- and physician-reported outcomes in the French National Registry of Facioscapulohumeral Dystrophy
Source: Orphanet J Rare Dis. 2022 Mar 2;17:96. doi: 10.1186/s13023-021-01793-6 (PMC8890461; doi:10.1186/s13023-021-01793-6)

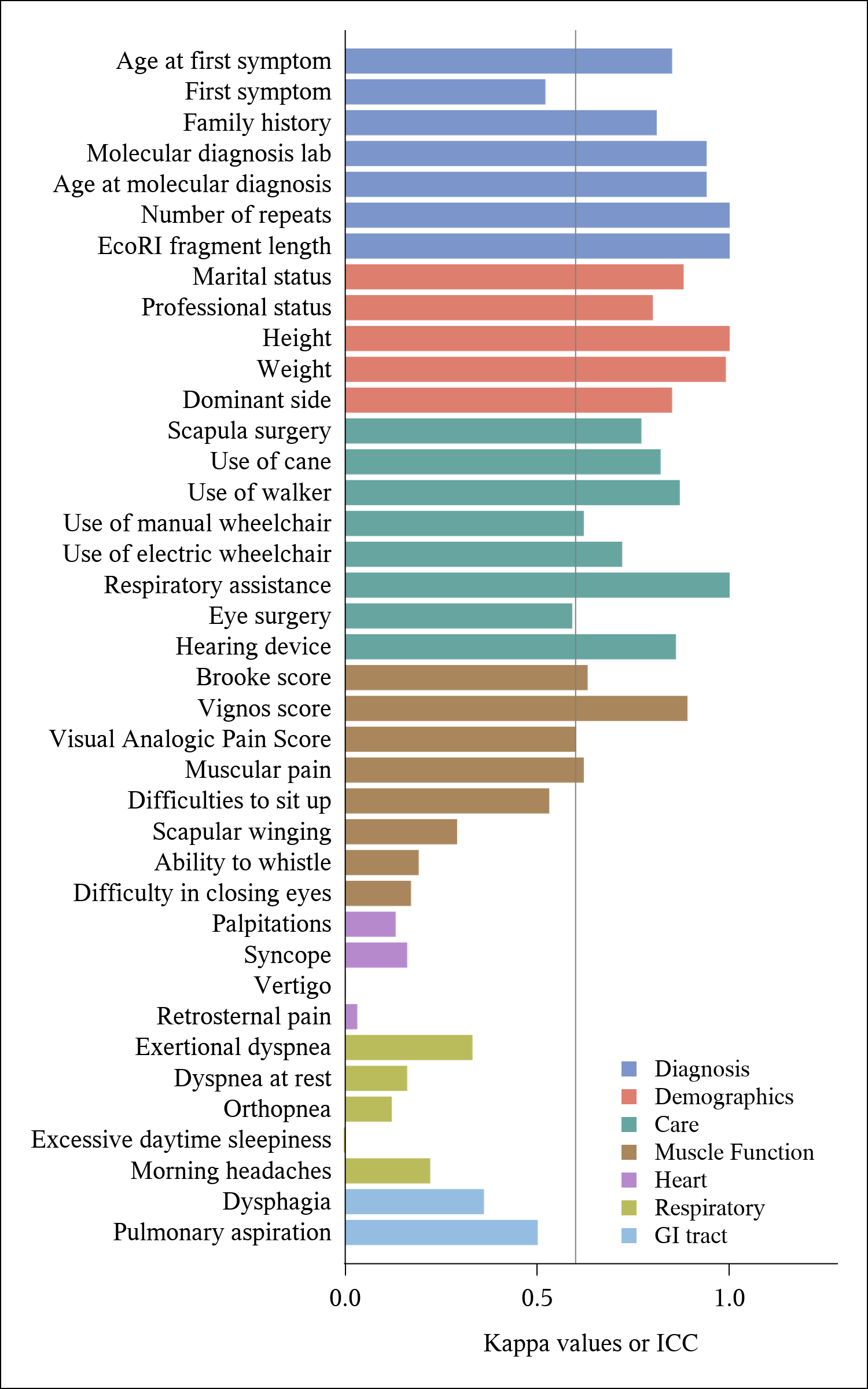

Supplement: Supplementary file 1 — Additional file 1. Figure S1: Agreement (in Kappa or ICC values) between item answers in the SRQ and the CEF in the younger subgroup of the cohort (< 65 years; N = 209). [file 13023_2021_1793_MOESM1_ESM.png]

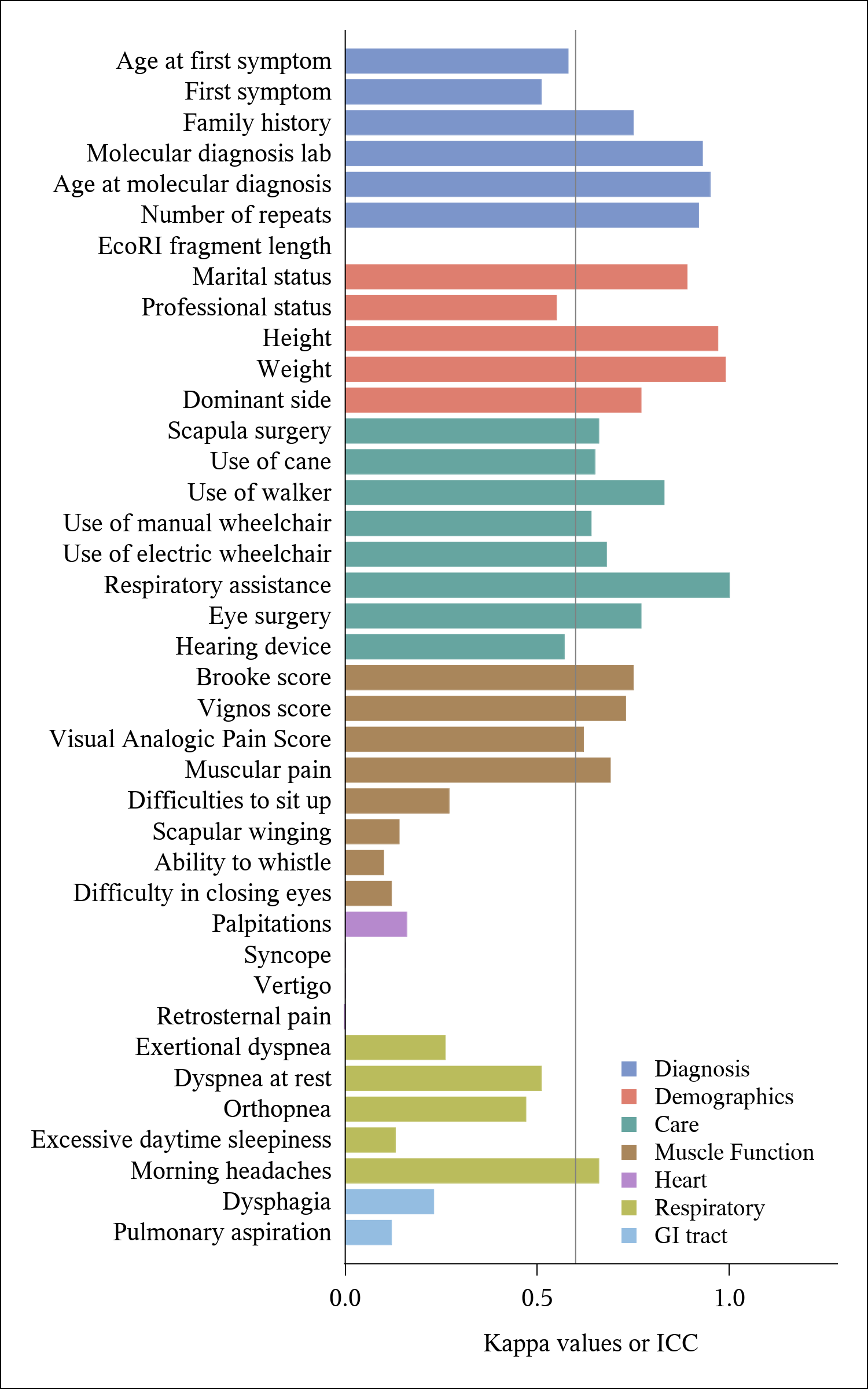

Supplement: Supplementary file 2 — Additional file 2. Figure S2: Agreement (in Kappa or ICC values) between item answers in the SRQ and the CEF in the older subgroup of the cohort (≥ 65 years, N = 209). [file 13023_2021_1793_MOESM2_ESM.png]

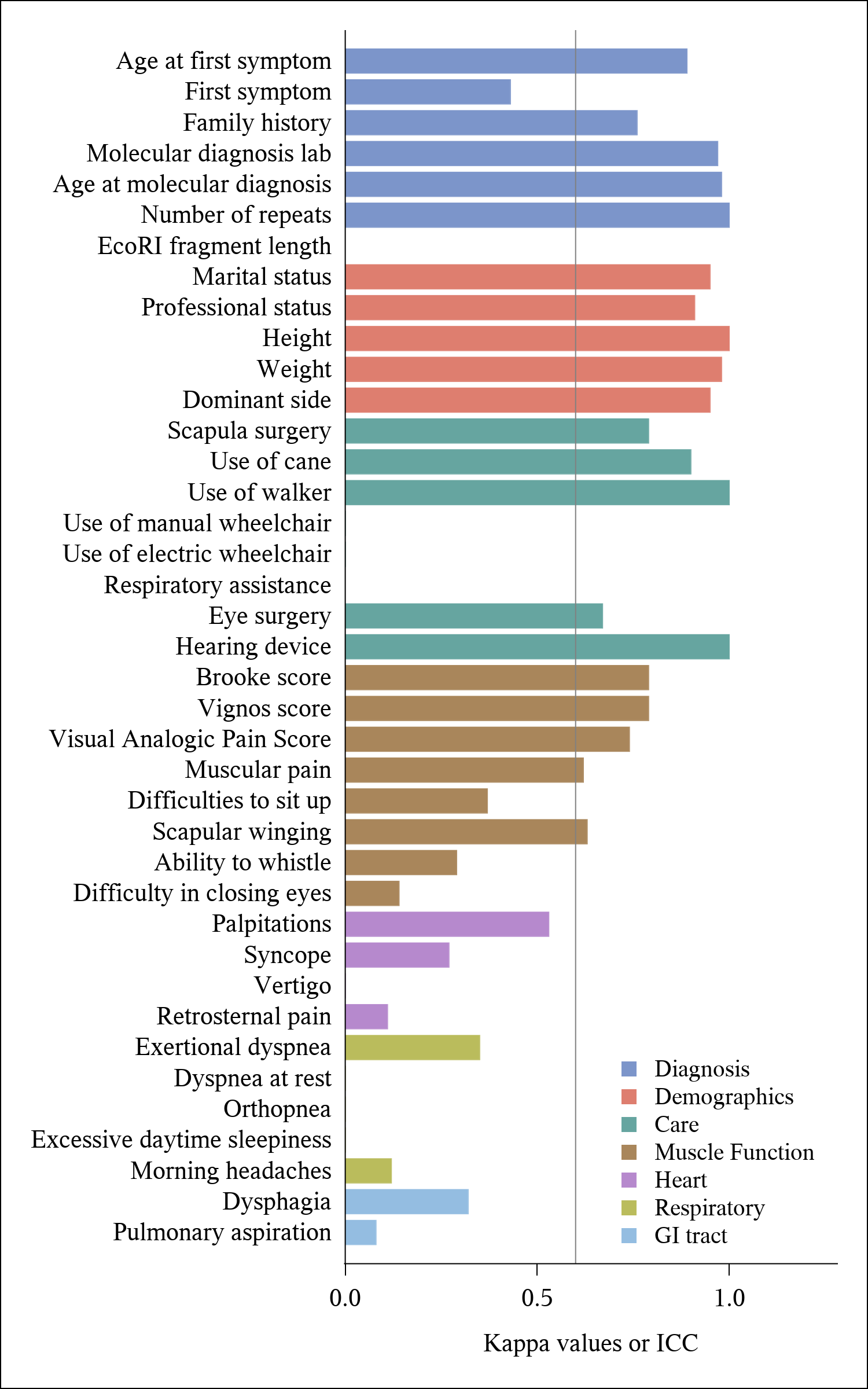

Supplement: Supplementary file 3 — Additional file 3. Figure S3: Agreement (in Kappa or ICC values) between item answers in the SRQ and the CEF in the subgroup associated with less severe forms of the disease (CSS < 6, N = 72). [file 13023_2021_1793_MOESM3_ESM.png]

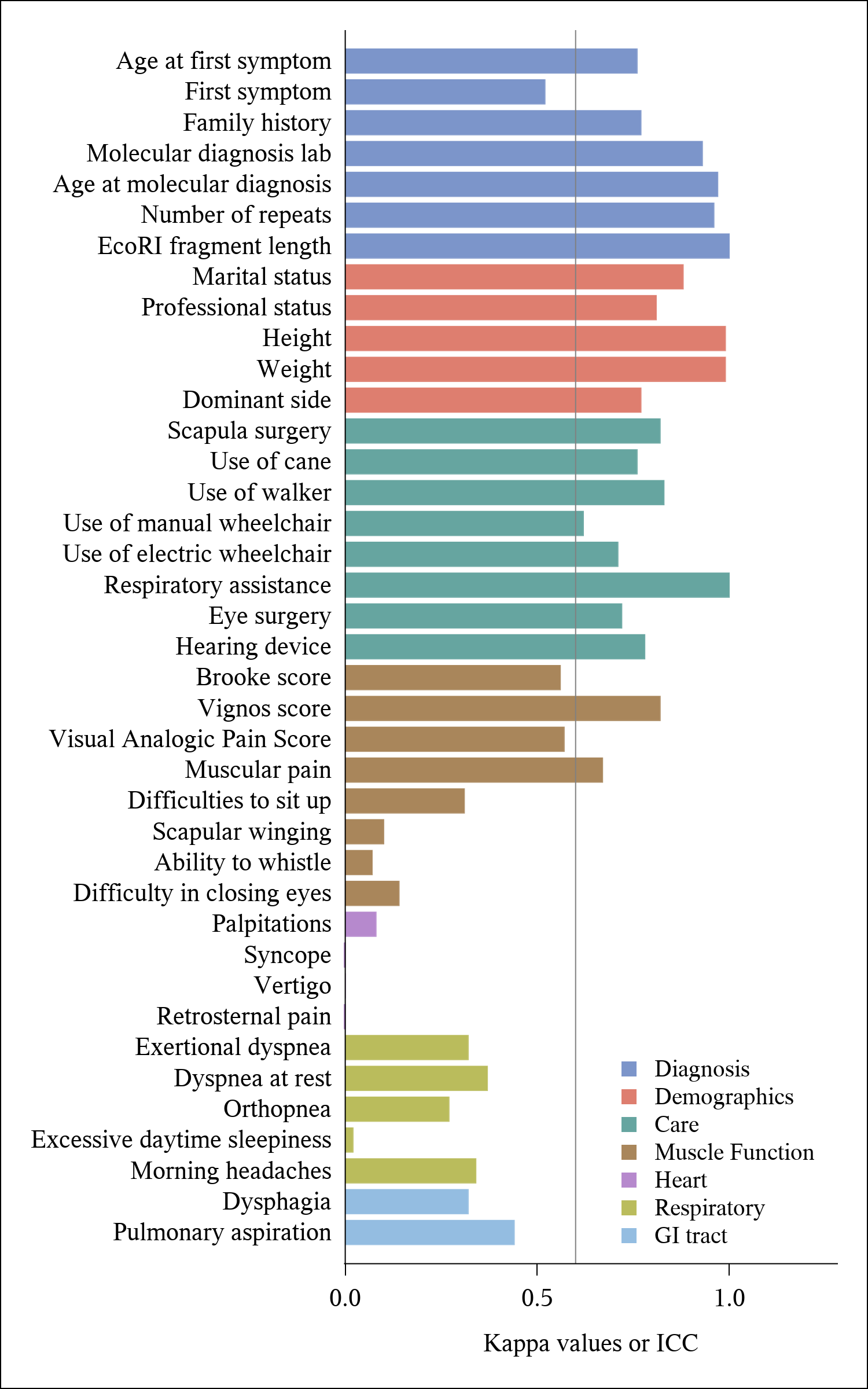

Supplement: Supplementary file 4 — Additional file 4. Figure S4: Agreement (in Kappa or ICC values) between item answers in the SRQ and the CEF in the subgroup associated with more severe forms of the disease (CSS ≥ 6; N = 165). [file 13023_2021_1793_MOESM4_ESM.png]

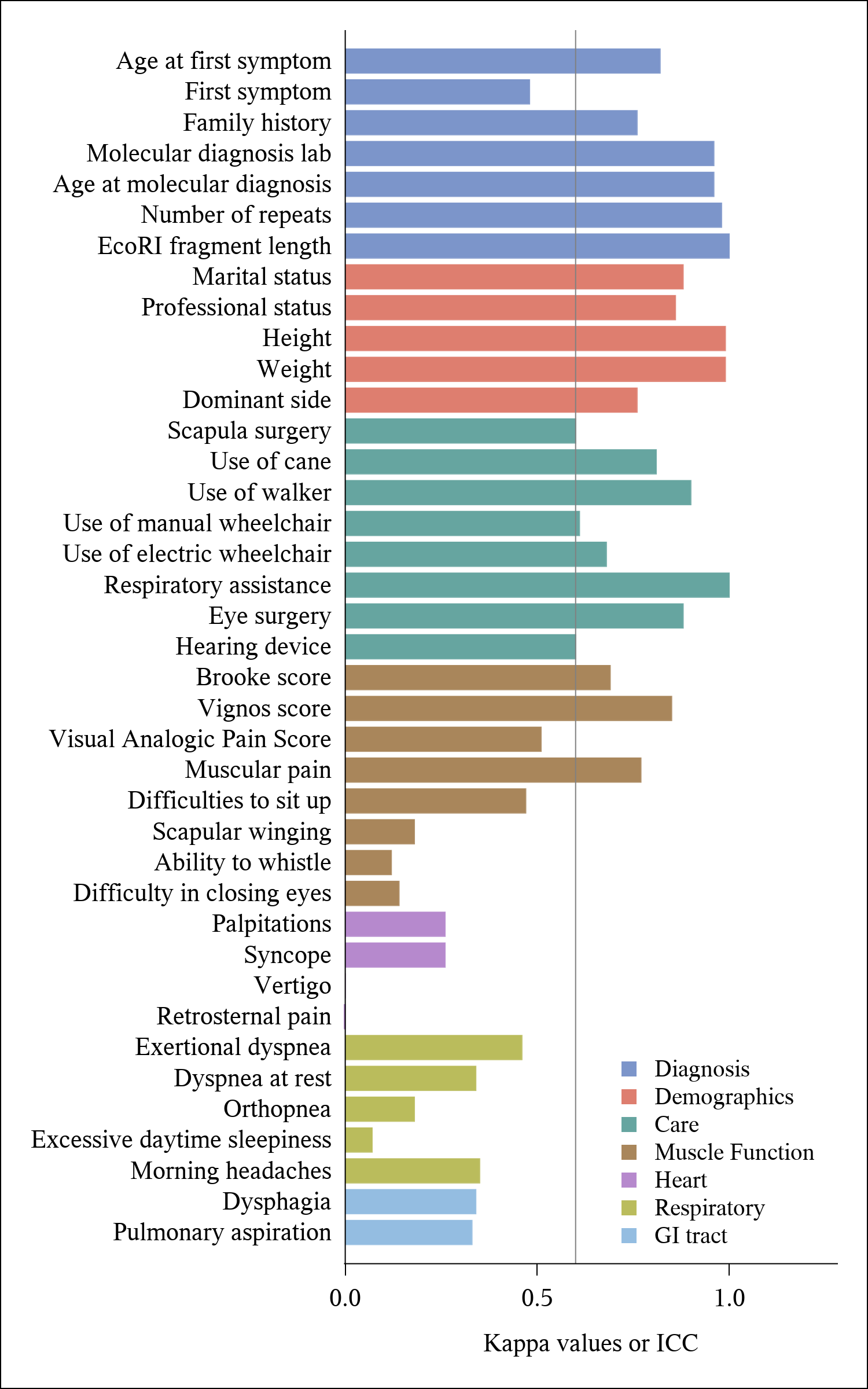

Supplement: Supplementary file 5 — Additional file 5. Figure S5: Agreement (in Kappa or ICC values) between item answers in the SRQ and the CEF in the subgroup associated with men. [file 13023_2021_1793_MOESM5_ESM.png]

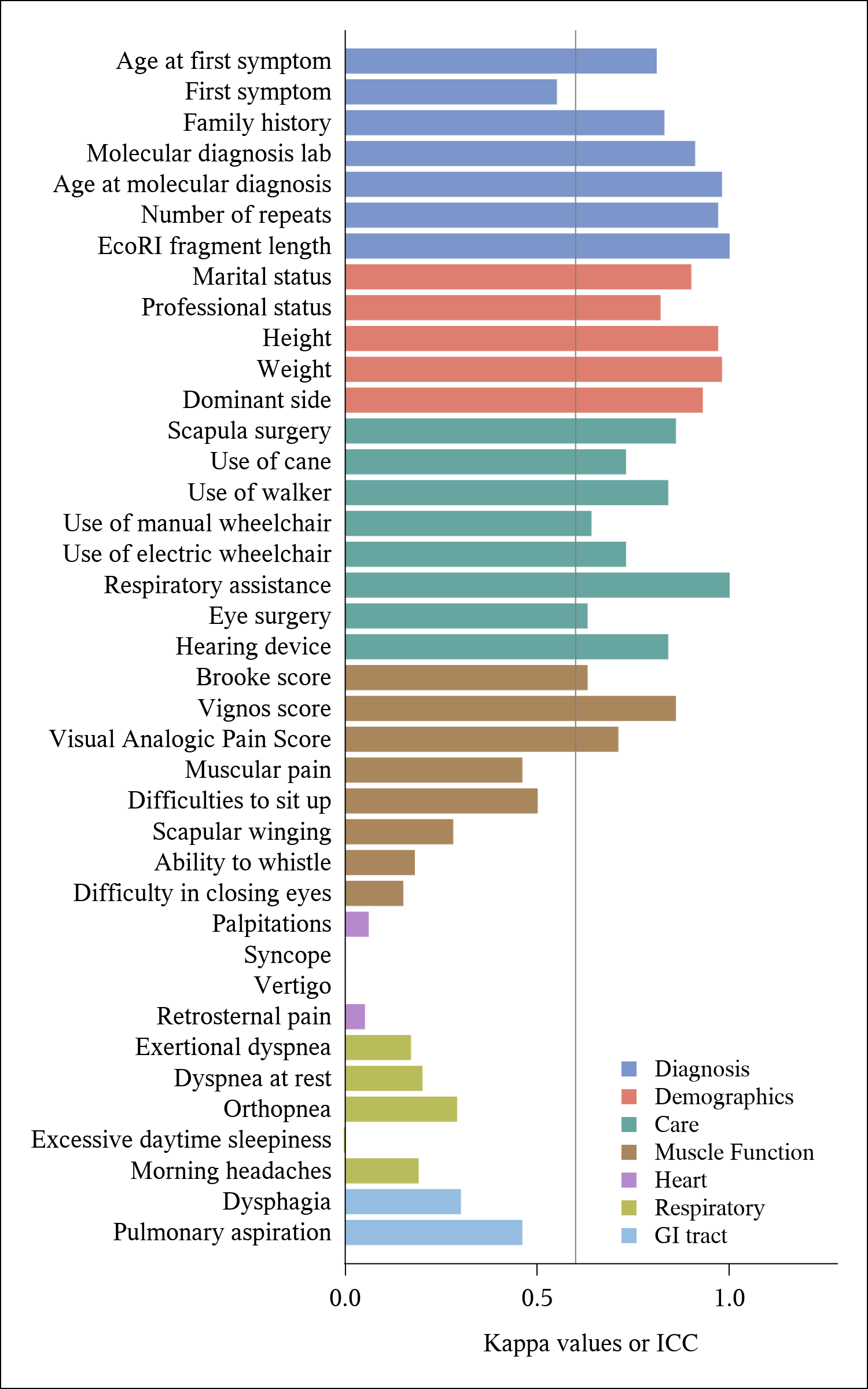

Supplement: Supplementary file 6 — Additional file 6. Figure S6: Agreement (in Kappa or ICC values) between item answers in the SRQ and the CEF in the subgroup associated with women. [file 13023_2021_1793_MOESM6_ESM.png]

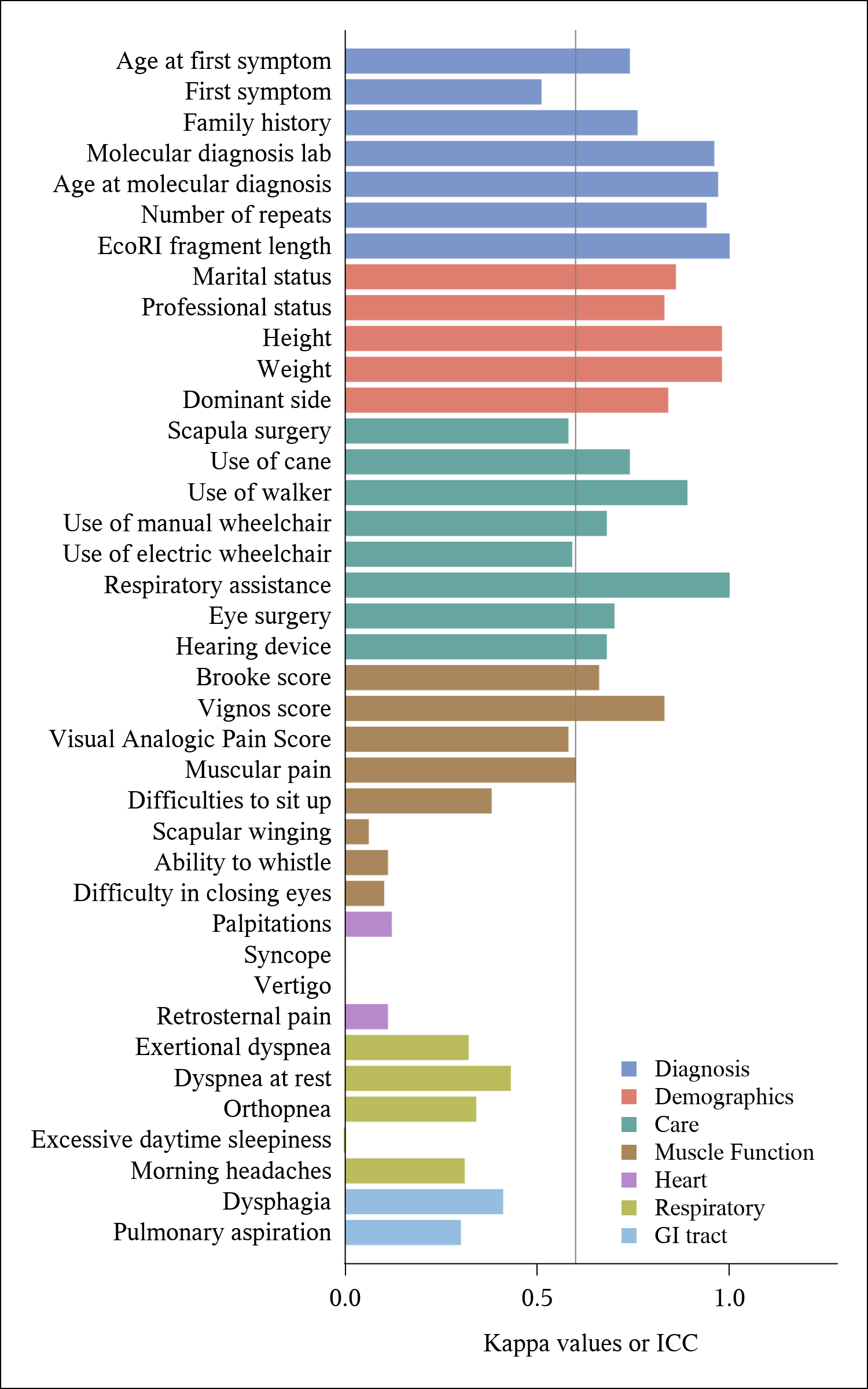

Supplement: Supplementary file 7 — Additional file 7. Figure S7: Agreement (in Kappa or ICC values) between item answers in the SRQ and the CEF in the subgroup associated with pre- French secondary education degree. [file 13023_2021_1793_MOESM7_ESM.png]

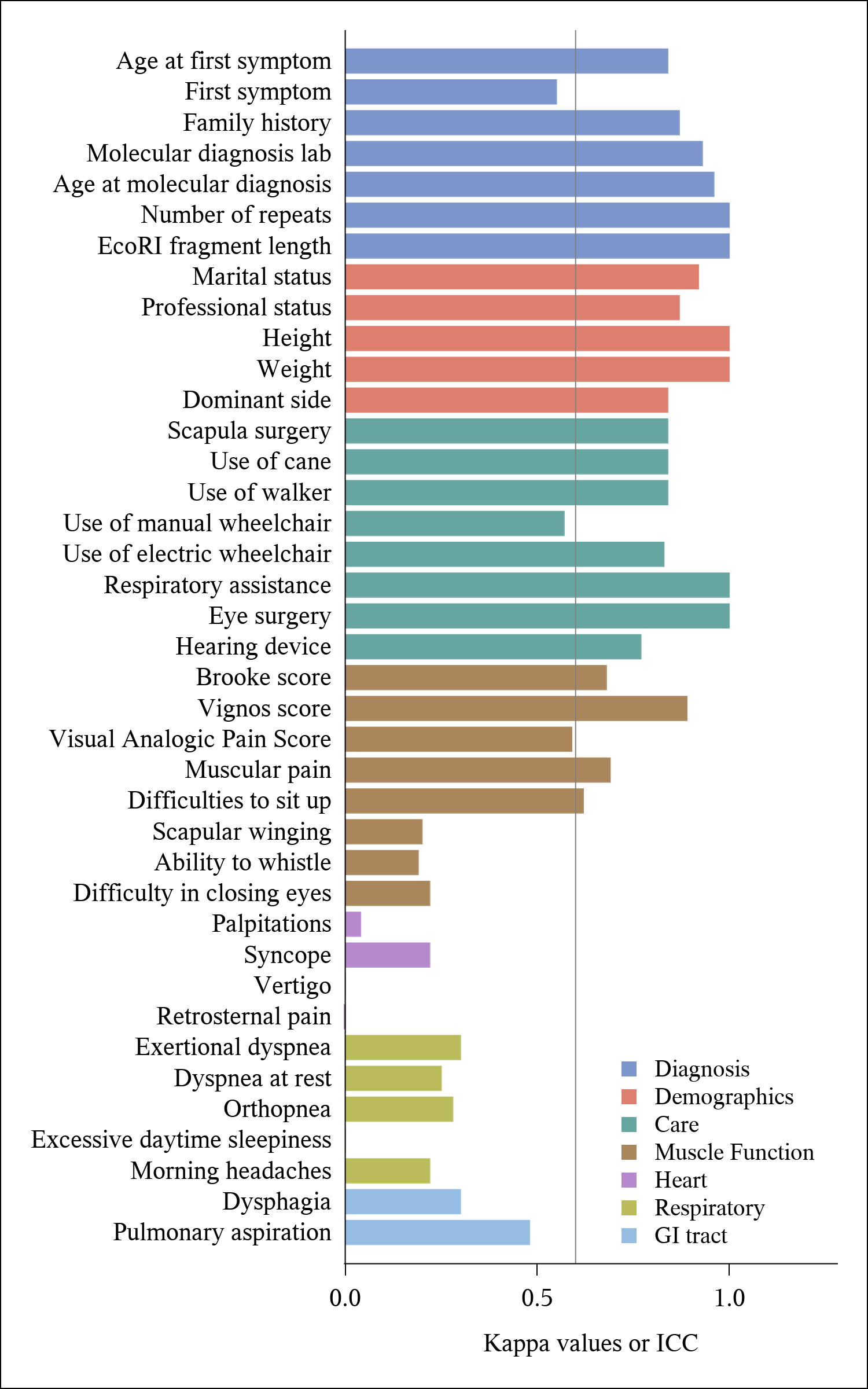

Supplement: Supplementary file 8 — Additional file 8. Figure S8: Agreement (in Kappa or ICC values) between item answers in the SRQ and the CEF in the subgroup associated with post- French secondary education degree. [file 13023_2021_1793_MOESM8_ESM.png]
